# Supplementary material for: Causal interplay between lactose intolerance and gut microbiota: a combined bidirectional Mendelian randomization and in vivo validation study
Source: Front Nutr. 2026 Jun 1;13:1803337. doi: 10.3389/fnut.2026.1803337 (PMC13265576; doi:10.3389/fnut.2026.1803337)
Supplement: Supplementary file 1 [file Data_Sheet_1.zip › supplementary materials/Forward/leave one out cross validation/ebi-a-GCST90027758.finngen_R12_E4_LACTONAS.pdf]

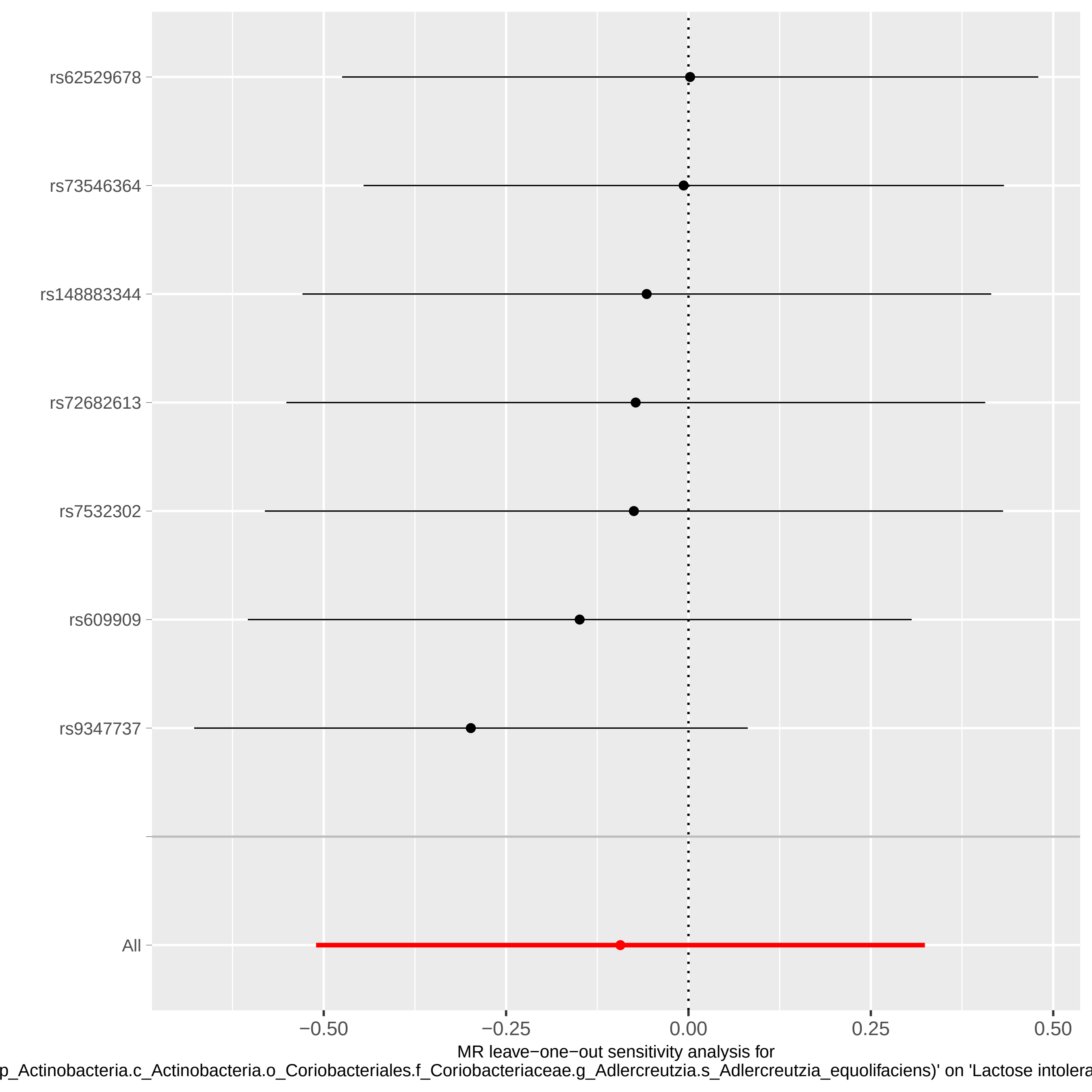
rs62529678
rs73546364
rs148883344
rs72682613
rs7532302
rs609909
rs9347737
All
−0.50
−0.25
0.00
0.25
0.50
MR leave−one−out sensitivity analysis for
p_Actinobacteria.c_Actinobacteria.o_Coriobacteriales.f_Coriobacteriaceae.g_Adlercreutzia.s_Adlercreutzia_equolifaciens)' on 'Lactose intolera
